# Reply to Guidolin et al. Comment on “Somnin et al. Study of Interactions Between Gadolinium-Based Contrast Agents and Collagen by Taylor Dispersion Analysis and Frontal Analysis Continuous Capillary Electrophoresis. *Pharmaceuticals* 2024, *17*, 1633”

**DOI:** 10.3390/ph18091284

**Published:** 2025-08-28

**Authors:** Chutintorn Somnin, Joseph Chamieh, Laurent Leclercq, Christelle Medina, Olivier Rousseaux, Hervé Cottet

**Affiliations:** 1 IBMM, University of Montpellier, CNRS, ENSCM, 34095 Montpellier, France; chutintorn.somnin@etu.umontpellier.fr (C.S.); laurent.leclercq@umontpellier.fr (L.L.); 2 GUERBET, Research and Innovation, 16 rue Jean Chaptal, 93600 Aulnay Sous Bois, France; christelle.medina@guerbet.com (C.M.); olivier.rousseaux@guerbet.com (O.R.)

We understand your surprise regarding our data showing a GBCA retention on Amicon^®^ filters. As we mentioned in our previously published article [1], we were also surprised by this finding. But these are the results we obtained, and this is why we rapidly discarded the use of filtration in our study to avoid any loss of GBCA and any bias in the interaction study.

Even more surprising is that you did not measure any significant GBCA retention on Amicon^®^ Ultra-0.5 mL filters in your last experiments [2], performed in very similar experimental conditions to article [1]. We have no clear explanation for that, except that it could be dependent on the specific batch of the filters. In paper [1], we tried filtration on an Amicon^®^ Ultra-0.5 mL device at two different concentrations (2.5 and 5.0 mM), even if only one concentration was presented in the paper. We report here the results obtained at the two different concentrations (see Figure 1).

Regarding the use of Taylor dispersion analysis for measuring GBCA concentration, it should be noted that, in Figure 5 and Table S1 of Ref. [1] and in Figure 1 of the present letter, we used TDA as a simple UV spectrophotometer (UV detection through the capillary) by measuring the absorbance at 200 nm on the plateau, before and after filtration. UV spectrometry is a well-known and adopted technique for measuring any UV absorbing substance in solutions such as GBCA. Doing the measurements in a capillary presents the advantages of using low sample volumes.

Retention during ultrafiltration is quite common [3] and can alter the exact composition of the filtrated solution. Even if it is more often observed for hydrophobic components, hydrophilic interactions, e.g., via H-bonding and van der Waals interactions, are also possible for hydrophilic solutes. Regarding the non-specific retention of GBCA on filters, we would like to share recent ultrafiltration data (courtesy of Prof. Uwe Karst, University of Munster [4]) that also demonstrate the strong retention of GBCA on Amicon^®^ filters (see Figure 2). Even if these results were obtained at much lower GBCA concentrations (in the sub-µM range), recovery ~90% for Prohance down to ~60% for Dotarem were observed. Recovery could be significantly increased after three successive rinsings with deionized water, while it was almost complete for ProHance (see Figure 3).

Regarding the study of the interaction of GBCA with collagen, taking the interaction parameter given in [5] for Dotarem as an example (see Table 1 in [5], log *b* = 2.54; *Q* = 7.1 × 10^−6^ mol/g with *b* the binding site constant and *Q* the maximum amount of GBCA adsorbed onto collagen), Figure 4 displays the free GBCA concentration at equilibrium in a mixture containing 75 mg/mL of collagen as a function of the introduced GBCA concentration, [GBCA]_tot_. The blue curve corresponds to the free GBCA concentration if there were no interactions and the red line corresponds to the free concentration taking the above parameters of interaction. This figure clearly shows that the decrease in GBCA concentration, due to the interaction for such a very low binding constant and stoichiometry, is limited to typically about −5% to −10% of the introduced concentration. This change in concentration is difficult to determine precisely and requires an analytical method with high sensitivity. It is also clear that the determination of the free GBCA concentration can be easily perturbed by retention on the filter. In our study without the use of filtration [1], we did not see any decrease in the GBCA concentration after the addition of collagen in the mixture, either by TDA or by frontal continuous capillary electrophoresis (FACCE). This is why we have concluded that there is no significant (or measurable) interaction between GBCA and collagen. We agree that there is still the possibility that TDA and FACCE are not sensitive enough to be able to determine very weak interactions. Sensitivity, LOD, and LOQ figures of merit are given in Table 1 for frontal TDA and different detection modes (reported from Ref. [6]).

Finally, we would like also to mention that FACCE was recently successfully implemented to study the interaction of GBCA with lysozyme [7]. Cooperative interactions were observed with the following increasing order of the binding constant, Multihance < Magnevist < Dotarem, while Elucirem did not interact with lysozyme.

In conclusion, we did observe the retention of GBCA on Amicon^®^ Ultra-0.5 mL filters [1], in agreement with recently reported results obtained by other groups [4]. The reason why the authors did not observe any retention on Amicon^®^ Ultra-0.5 mL filters in similar conditions [2] is not clear, but could be related to differences in filter batches. We rationally discarded the use of filtration in our interaction studies between GBCA and collagen or lysozyme [1,7] to avoid any impact of the filtration on the interaction studies. We did not see any significant interaction between GBCA and collagen using TDA and FACCE [1], while we did observe and quantify cooperative interactions between GBCA and lysozyme [7]. It is true that the absence of interaction observed in Ref. [1] is relative to the sensitivity of detection in the techniques that we used (FACCE and frontal TDA with UV and LEDIF detections). Demonstrating the absence of interaction between two entities is more difficult to prove than determining interaction parameters when they are measurable. When no interaction is observed with a given technique, we can only conclude that there is no measurable interaction relative to the sensitivity of detection in this technique.

**Materials and Methods**
**Experimental procedure of retention study using frontal TDA** 

A total of 500 µL of GBCA solution (2.5 mM or 5.0 mM) in 10 mM Tris-HCl pH 7.4 was added into an Amicon^®^ Ultra-0.5 mL centrifugal filter (MWCO 10kDa, Merck Millipore, Darmstadt, Germany, Ref. no. UFC501024, Lot no. 0000238696). After that, the solution was centrifuged at 12,000 rpm for 3 min at 4 °C. Almost all of the solution passed through the filter, but only 100 µL of sample was collected to analyze using frontal TDA. TDA was performed in a bare fused-silica capillary (Polymicro technologies, Phoenix, USA) with a 50 µm i.d. × 65 cm total length. UV detection windows (200 nm and 270 nm) were positioned at 56.5 cm from the inlet. Capillaries were conditioned before analysis by flushing (at 1 bar) with water for 10 min, followed by the background electrolyte (BGE) for 10 min. The BGE was 10 mM Tris-HCl at pH 7.4. Samples were continuously introduced at 100 mbar from the inlet side of the capillary. Between runs, the capillary was rinsed by flushing with the BGE for 5 min. The temperature of the carousel holding the BGE and sample vials was controlled using a thermostatic bath set at 37 °C (Instrumat, Moirans, France). Signal acquisition was carried out using Chemstation software (B.04.02 SP1), then exported to Microsoft excel for subsequent data treatment.

**Experimental procedure of recovery study after ultrafiltration using ion chromatography coupled to ICP-MS (courtesy of Prof. Uwe Karst** [4]**)** 

Separate 200 nmol/L stock solutions of the GBCAs were prepared in doubly distilled water. For the ultrafiltration experiment, 500 µL of 200 nmol/L GBCA solution was centrifuged using Amicon^®^ Ultra-0.5mL centrifugal filters (0.5 mL sample volume, 10 kDa molecular weight cutoff, regenerated cellulose membrane) for 20 min at 14,000× *g* and 4 °C. The filter with the residual solution was placed in a clean microcentrifuge tube, 100 µL of doubly distilled water was added, and the centrifugation was repeated under identical conditions. This washing step was conducted a total of three times. The method for chromatographic separation of GBCAs is based on the work of Macke et al. [8] and employs a CF-Gd-01 (50 × 4 mm, Elemental Scientific, Omaha, NE, USA) anion exchange column.

## Figures and Tables

**Figure 1:**
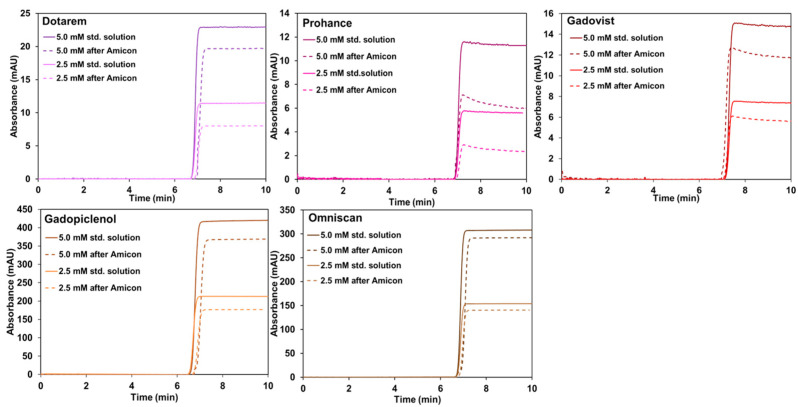
Frontal taylorgrams before and after filtration using an Amicon^®^ Ultra-0.5 mL device, MWCO of 10 kDa, showing the retention of GBCA (2.5 mM and 5.0 mM). Experimental conditions: fused silica capillary of 65 cm total length (56.5 cm to UV detector) × 50 µm i.d. eluent: 10 mM tris buffer (pH 7.4). Mobilization pressure: 100 mbar. UV detection at 200 nm. Sample volume: 60 µL. TDA experiments were performed at 37 °C. Centrifugation conditions: 12,000 rpm for 3 min at 4 °C.

**Figure 2:**
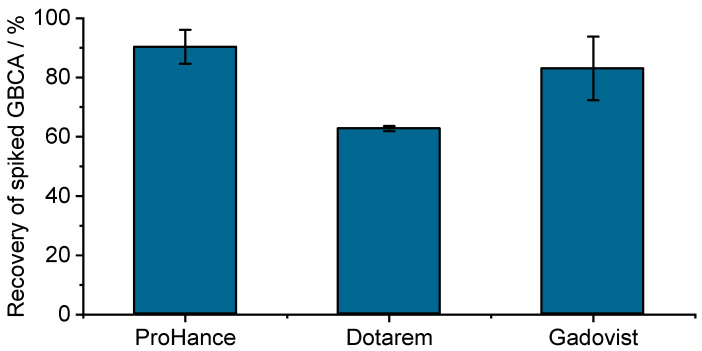
Recovery of GBCA solutions after ultrafiltration on Amicon^®^ Ultra-0.5 mL centrifugal filters. A total of 500 µL of 200 nM GBCA solution was prepared in doubly distilled water and filtrated using Amicon^®^ Ultra centrifugal filters (0.5 mL sample volume, 10 kDa molecular weight cutoff, regenerated cellulose membrane) for 20 min at 14,000× *g* and 4 °C. GBCA concentrations in the filtrate solutions were determined by ion chromatography coupled to ICP-MS (courtesy of Prof. Uwe Karst, University of Munster [4]). Error bars represent the standard deviation of triplicate analysis.

**Figure 3:**
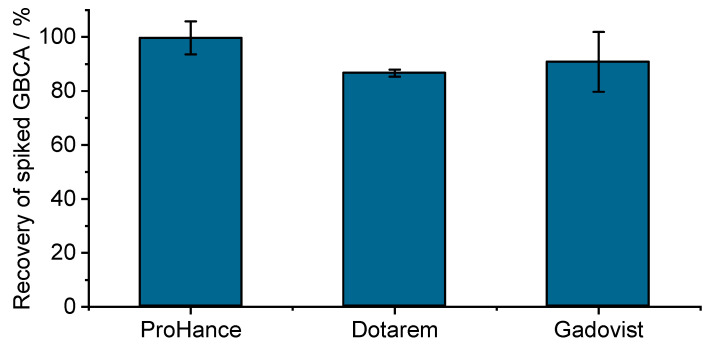
Combined recovery of GBCA solutions after ultrafiltration on Amicon^®^ Ultra-0.5 mL centrifugal filters and three rinsing steps with water. A total of 500 µL of 200 nM GBCA solution was prepared in doubly distilled water and filtrated using Amicon^®^ Ultra centrifugal filters (0.5 mL sample volume, 10 kDa molecular weight cutoff, regenerated cellulose membrane) for 20 min at 14,000× *g* and 4 °C. Filters were next washed three times with 100 µL of distilled water. GBCA concentrations in the filtrate + washing solution were determined by ion chromatography coupled to ICP-MS (courtesy of Prof. Uwe Karst, University of Munster [4]). Error bars represent the standard deviation of triplicate analysis.

**Figure 4:**
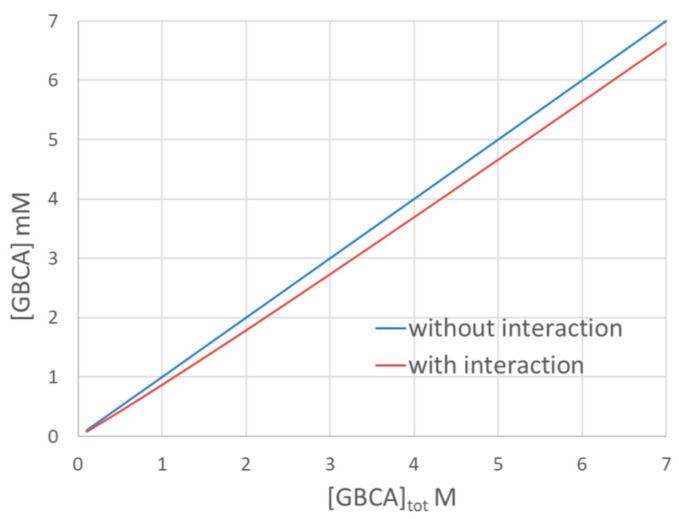
Calculated free GBCA concentration as a function of the introduced concentration for Dotarem using interaction parameters given in Ref. [5] in a solution containing 75 mg/mL of collagen type I. log *b* = 2.54; *Q* = 7.1 × 10^−6^ mol/g with *b* the binding site constant and *Q* the maximum amount of GBCA adsorbed onto collagen.

**Table 1:** Sensitivity, LOD, and LOQ obtained by TDA using UV and LEDIF detections from Ref. [6].

Sample	BGE	UV Detection at 200 nm and 270 nm			LEDIF λ_ex_ 275 nm λ_collection_ 300–450 nm and λ_collection_ 300–330 nm		
		Slope (AU M^−1^)	LOD ^e^ (mM)	LOQ ^e^ (mM)	Slope (RFU mM^−1^)	LOD ^e^ (mM)	LOQ ^e^ (mM)
Gd-DTPA-BMA (Omniscan^®^)	Water	57.7 ^a^ n.d. ^b^	0.10 ^a^ n.d. ^b^	0.32 ^a^ n.d. ^b^	0.058 ^c^ 0.033 ^d^	0.71 ^c^ 1.72 ^d^	2.36 ^c^ 5.72 ^d^
Gd-BOPTA (MultiHance^®^)	Water	51.2 ^a^ n.d. ^b^	0.18 ^a^ n.d. ^b^	0.61 ^a^ n.d. ^b^	n.d. ^c^ n.d. ^d^	n.d. ^c^ n.d. ^d^	n.d. ^c^ n.d. ^d^
Gd-HP-DO3A (Prohance^®^)	Tris-HCl	1.96 ^a^ n.d. ^b^	0.84 ^a^ n.d. ^b^	2.81 ^a^ n.d. ^b^	0.171 ^c^ 0.075 ^d^	0.29 ^c^ 0.76 ^d^	0.97 ^c^ 2.52 ^d^
Gd-BT-DO3A (Gadovist^®^)	Tris-HCl	2.65 ^a^ n.d. ^b^	0.70 ^a^ n.d. ^b^	2.35 ^a^ n.d. ^b^	0.102 ^c^ 0.053 ^d^	1.50 ^c^ 0.39 ^d^	5.00 ^c^ 1.28 ^d^
Gd-DOTA (Dotarem^®^)	Tris-HCl	2.63 ^a^ n.d. ^b^	0.22 ^a^ n.d. ^b^	0.72 ^a^ n.d. ^b^	0.202 ^c^ 0.094 ^d^	0.56 ^c^ 1.06 ^d^	1.87 ^c^ 3.52 ^d^
Gd-PCTA D2 (Elucirem^®^)	Tris-HCl	85.6 ^a^ 16.3 ^b^	0.12 ^a^ 0.06 ^b^	0.40 ^a^ 0.20 ^b^	0.129 ^c^ 0.063 ^d^	0.43 ^c^ 0.88 ^d^	1.38 ^c^ 2.94 ^d^

^a^ UV at 200 nm, ^b^ UV at 270 nm, ^c^ LEDIF using λ_collection_ 300–450 nm, ^d^ LEDIF using λ_collection_ 300–330 nm. ^e^ LOD and LOQ were calculated by 3 × *s_y/x_*/*a* and 10 × *s_y/x_*/*a*, respectively, where *s_y/x_* is the regression standard deviation and *a* is the slope of the calibration curve. From Ref. [6]. n.d. = not detected.

## Data Availability

Data is contained within the article.
